# Climate Change and Topography Drive the Expansion of *Betula ermanii* in the Alpine Treeline Ecotone of the Changbai Mountain

**DOI:** 10.1002/ece3.71368

**Published:** 2025-05-08

**Authors:** Yingyi Chen, Yongfeng Gu, Wen J. Wang, Lei Wang, Xiaodong Li, Shengwei Zong, Mai‐He Li, Zhengfang Wu, Hong S. He, Yu Cong, Ming Jiang

**Affiliations:** ^1^ Northeast Institute of Geography and Agroecology Chinese Academy of Sciences Changchun China; ^2^ Ecological Environment Monitoring and Scientific Research Center, SongLiao River Basin Ecological and Environment Administration Ministry of Ecology and Environment Changchun China; ^3^ Key Laboratory of Geographical Processes and Ecological Security in Changbai Mountains, Ministry of Education, School of Geographical Sciences Northeast Normal University Changchun China; ^4^ Shandong Key Laboratory of Eco‐Environmental Science for the Yellow River Delta Shandong University of Aeronautics Binzhou China; ^5^ Swiss Federal Institute for Forest, Snow and Landscape Research WSL Birmensdorf Switzerland; ^6^ School of Life Science Hebei University Baoding China; ^7^ School of Natural Resources University of Missouri Columbia Missouri USA

**Keywords:** Geodetector, global warming, mountain aspects, spatiotemporal distribution, treeline dynamics, trees expansion

## Abstract

Alpine treelines ecotones are critical ecological transition zones and are highly sensitive to global warming. However, the impact of climate on the distribution of treeline trees is not yet fully understood as this distribution may also be affected by other factors. Here, we used high‐resolution satellite images with climatic and topographic variables to study changes in treeline tree distribution in the alpine treeline ecotone of the Changbai Mountain for the years 2002, 2010, 2017, and 2021. This study employed the Geodetector method to analyze how interactions between climatic and topographic factors influence the expansion of *Betula ermanii* on different aspect slopes. Over the past 20 years, *B. ermanii*, the only tree species in the Changbai Mountain tundra zone, had its highest expansion rate from 2017 to 2021 across all the years studied, approaching 2.38% per year. In 2021, *B. ermanii* reached its uppermost elevations of 2224 m on the western aspects and 2223 m on the northern aspects, which are the predominant aspects it occupies. We also observed a notable increase in the distribution of *B. ermanii* on steeper slopes (> 15°) between 2002 and 2021. Moreover, we found that interactions between climate and topographic factors played a more significant role in *B. ermanii*'s expansion than any single dominant factor. Our results suggest that the interaction between topographic wetness index and the coldest month precipitation (Pre_1_), contributing 91% of the observed variability, primarily drove the expansion on the southern aspect by maintaining soil moisture, providing snowpack thermal insulation which enhanced soil temperatures, decomposition, and nutrient release in harsh conditions. On the northern aspect, the interaction between elevation and mean temperature of the warmest month explained 80% of the expansion. Meanwhile, the interaction between Pre_1_ and mean temperature of the growing season explained 73% of the expansion on the western aspect. This study revealed that dominant factors driving treeline upward movement vary across different mountain aspects. Climate and topography play significant roles in determining tree distribution in the alpine treeline ecotone. This knowledge helps better understand and forecast treeline dynamics in response to global climate change.

## Graphical abstract

Over the past 20 years, the expansion rate of *Betula ermanii* in the alpine ecotone of Changbai Mountain varied with mountain aspects. We found that interactions between climate and topographic factors played a more significant role in *B. ermanii*'s expansion than any single dominant factor. Climate and topography play significant roles in determining tree distribution in the alpine treeline ecotone.

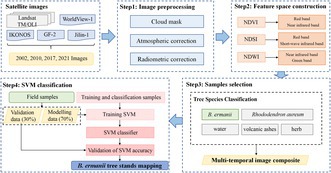

## 1 Introduction

The alpine treeline is the distribution boundary between closed forests and high mountain vegetation, and serves as a sensitive indicator of the impacts of climate change on forest ecosystems (Lu et al. 2021). Treeline trees have expanded to higher elevations in response to climate change, causing upward shifts of alpine treelines worldwide (Devi et al. 2020; He et al. 2023; Sigdel et al. 2024). However, some stable or even downward shifts in treelines occurring in harsh environmental conditions have been reported (Kullman 2007; Chhetri and Cairns 2015; Xu et al. 2020). Changes in treeline dynamics have broad ecological and societal implications, including shifts in biodiversity (Zhou, Zhang, et al. 2021), community taxonomic composition (Sanczuk et al. 2023) and trophic dynamics (García‐Valdés et al. 2020; Kumar and Khanduri 2024; Gupta et al. 2024), carbon dynamics (Schmeller et al. 2024), as well as correlations with social and population density (Thornton et al. 2021). Therefore, there is a need to better understand the importance of various factors that influence treeline dynamics to better predict vegetation responses to global warming and for preserving the ecological integrity of alpine ecotone biodiversity, both of which are essential for sustainable management (Cong et al. 2024).

Low temperatures are generally limiting factors for the recruitment and growth of treeline trees on a global scale (Körner 1999). Remarkably, approximately 70% of global treelines were observed to undergo upward migration over the period from 2000 to 2010 under global warming, with an average upward shift rate of 1.2 m/year (He et al. 2023). This upward shift could be partly explained by elevation‐dependent warming, where the rate of temperature increase was generally greater at higher elevations, thus accelerating tree growth in these areas (Pepin et al. 2022). However, regional evidence also indicated that due to precipitation limitations, tree growth at the treeline may not increase (Lyu et al. 2016; Camarero et al. 2021; Xie et al. 2024). In the Himalayas, Sigdel et al. (2018) observed upward shifts in the treelines in response to climate warming, but the shift rates were moderated by spring precipitation. This could be attributed to the fact that adequate precipitation and moisture may enhance tree growth rates and establishment within this limit (Camarero et al. 2021; Körner 1999). In addition, species interaction (Liang et al. 2016; Lyu et al. 2016), geomorphic processes (Macias‐Fauria and Johnson 2013) and topography (Elliott and Cowell 2015) have influenced treeline movements. Furthermore, topography has proven to be a crucial factor influencing the distribution of alpine plant species, particularly at local scales (Carmel and Kadmon 1999). Recent studies found that upward shifts of treeline were not uniform, with topographical variations contributing to local climate disparities that result in uneven expansion of treeline trees (Zhou, Mazepa, et al. 2021; Zheng et al. 2021). Moreover, the density of tree seedlings at Subarctic alpine treelines has been shown to vary with aspect (Kambo and Danby 2018).

Previous studies have mainly relied on field investigations to determine the spatiotemporal changes in treeline distribution in response to global warming (Qi et al. 2014; Wang et al. 2015; Gazol et al. 2022). While field investigations are effective for monitoring changes in treeline, they are mainly limited by sparse geographic coverage (He et al. 2023). Dendrochronology can reconstruct long‐term changes in the treeline (Du et al. 2017; Büntgen 2023); however, collecting tree cores and deriving tree‐ring data are time‐consuming and labor‐intensive. Recently, remote sensing images and historical maps have allowed us to analyze large‐scale treeline dynamics (Chhetri and Thai 2019; Mukhopadhyay et al. 2023; Garbarino et al. 2023). However, these data sources often have limitations in their spatial and temporal coverage. Although Landsat satellite data have been available since 1972, their spatial resolutions are too coarse to accurately identify trees (Beloiu et al. 2022). This limitation brings uncertainties and also leads to the treeline positions being identified at lower elevations than their actual locations, primarily due to the low spatial resolution (Wang et al. 2022). High‐resolution remote sensing images combined with field data can overcome this limitation and accurately reflect the forest distribution (Skurikhin et al. 2016; Beloiu et al. 2022; Xie et al. 2024).

In previous studies, climate change was found to be the primary factor driving the upward movements of treelines (Arekhi et al. 2018; Devi et al. 2020; Vacek et al. 2023). There have been substantial efforts in recent years to derive the causal effects of factors such as thermal variations (Xue et al. 2020; Xie et al. 2024), moisture availability (Lange et al. 2020; Singh et al. 2024), biotic interactions (Liang et al. 2016), and topographical characteristics (Frei et al. 2023) on treeline dynamics. However, at high elevations, the upward shift of the treeline mainly occurred in favorable microhabitats (Frei et al. 2023), which were influenced by both climatic and topographical factors. Those studies have reflected the influence of different factors on treeline dynamics, but not the interaction between various factors affecting treeline variation. Thus, understanding the combined or interactive effects of climate changes and topographical factors on the distribution of treeline tree species remains a challenge, and the role of topographical patterns and the limiting factors is still unclear (Tourville et al. 2023). Here, we provide a geographical detector model assessment of the combined effects of climatic and topographic factors on treeline tree species in the Changbai Mountain. We selected this method because it has previously been shown to effectively reveal the interactions and degrees of influence among variables, thus providing a more robust assessment of environmental impact on treeline distribution (Yan et al. 2023). Unlike traditional regression‐based methods, geographical detectors can effectively capture nonlinear interactions and spatial heterogeneity (Chen et al. 2023), offering a nuanced understanding of environmental influences on treeline patterns.

In this study, to better monitor the treeline dynamic trends, we analyze the spatiotemporal patterns of the alpine treeline species *Betula ermanii* in the Changbai Mountain in the past 20 years. We combine high‐resolution satellite imagery with local climatic and topographic variables to investigate the temporal dynamics of this tree species in the alpine ecotone. Our aim is to use the Geodetector method to detect the dominant factors driving the expansion of *B. ermanii*, the only tree species in the Changbai Mountain tundra zone. We hypothesize that: (i) the expansion rate of *B. ermanii* in the alpine treeline ecotone of Changbai Mountain varies with mountain aspect slopes, and (ii) multifactor determination (e.g., climate change and topography) drives the expansion of *B. ermanii* in the alpine treeline ecotones.

## 2 Materials and Methods

### 2.1 Study Area

**FIGURE 1:**
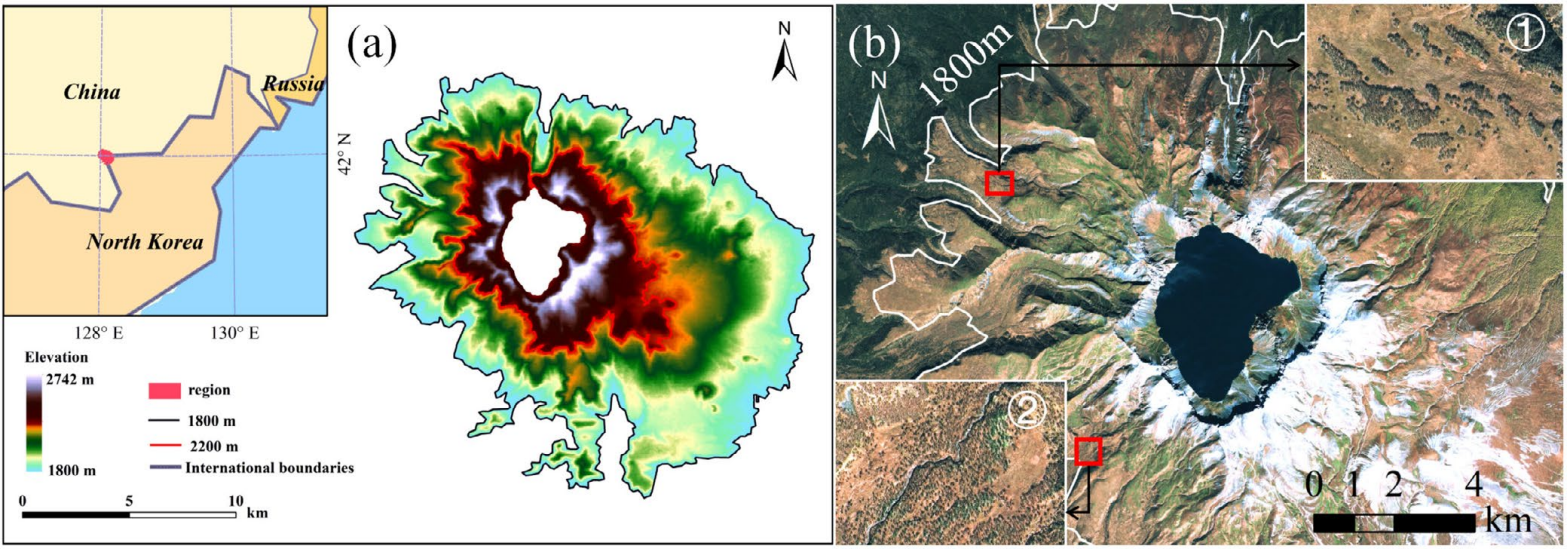
Location of the study area in northeastern China, at the border with North Korea: (a) Gradient colors represent elevation changes, with the study area located above 1800 m and (b) remote sensing image of the study area, with ① and ② marking the locations of the *B. ermanii* forest, highlighted to show its appearance in the imagery.

The Changbai Mountain (41.697°–42.422° N, 127.715°–128.280° E) is the highest peak in northeast China, located at the border to North Korea (Figure 1). The climate is a temperate continental climate characterized by low temperature and intense precipitation (Zong et al. 2014). The annual mean temperature ranges from −7.3°C to 4.9°C and annual precipitation ranges from 800 to 1800 mm, with elevation ranging from 713 to 2691 m above sea level (Du et al. 2017). The Changbai Mountain has four main vegetation zones along the altitudinal gradients: mixed coniferous broad‐leaved forests distributed from 740 to 1100 m a.s.l., coniferous forests from 1100 to 1700 m a.s.l., birch (*B. ermanii*) forests from 1700 to 1950 m a.s.l., and tundra zone above 2000 m a.s.l. (Yu et al. 2014). The area chosen for this study is located in the *B. ermanii* treeline ecotone between 1800 and 2200 m a.s.l., transitioning from close canopy forests to open areas with trees in scattered distributions of small patches. Over the past few decades, *B. ermanii* trees have encroached on the alpine tundra zone, leading to the expansion of the treeline ecotone (Zong et al. 2014). The study area is undisturbed by anthropogenic activity because of its remoteness and high elevation (Cong et al. 2022). Therefore, it provides an excellent opportunity to study *B. ermanii* tree's locations. In our study, we regarded the area between 1800 and 2200 m a.s.l. in the Changbai Mountain as the study area (Figure 1). However, since the eastern aspect in North Korea cannot be verified, we excluded this area from the study.

### 2.2 Data Source and Preprocessing

Landsat TM/OLI images provided by the United States Geological Survey (USGS) (http://earthexplorer.usgs.gov/) are used for the study. A total of 20 Landsat images (14 Landsat‐5 TM and 6 Landsat‐8 OLI), Path/Row 116/031 acquired from 2002 to 2014, are used to cover the study area (Table 1). The high‐resolution satellite images data including IKONOS, WorldView‐1, GaoFen‐2 (GF‐2) and Jilin‐1 data (https://www.kosmos‐imagemall.com/) were also used to obtain more accurate tree location data. We collected satellite images at frequent intervals, ensuring a maximum gap of 30 days between acquisitions to maintain temporal consistency. We also selected images from the end of the growing season (September and October) and averaged them to derive annual datasets, reducing variability from phenological differences and minimizing acquisition angle impacts. We selected a high‐resolution digital elevation model (DEM, 5 m) derived from the panchromatic remote‐sensing instrument for stereo mapping (PRISM) sensor attached to the advanced land observing satellite (ALOS). The DEM was resampled from 5 to 50 m resolution using the nearest‐neighbor method in ArcGIS 10.2. Meteorological data, including monthly precipitation and monthly temperature (maximum, minimum, and mean), were initially available at a 1 km spatial resolution and were derived from the National Earth System Science Data Center of China (http://www.geodata.cn) and CHELSA (https://chelsa‐climate.org). These data were downscaled to a 50 m resolution using multiple linear regression (MLR) (Kostopoulou et al. 2007), incorporating slope, elevation, and aspect from the DEM to improve the accuracy of climate data interpolation at finer spatial scales. The downscaled data were then validated using growing season temperature observations from 2015 to 2017 (Wang et al. 2019), achieving an RMSE of 0.94 and an *R* ^2^ of 0.90.

**TABLE 1:** List of satellite images used in this study.

Satellite	Sensor	Scene	Acquired date	Resolution	Cloud cover (%)
Landsat	Thematic Mapper (TM)	LT51160312001258BJC00	9/15/2001	30 m	0
		LT51160312002261BJC01	9/18/2002		
		LT51160312004267BJC00	9/23/2004		
		LT51160312005125BJC00	9/10/2005		
		LT51160312009280HAJ00	10/7/2009		
		LT51160312010267IKR00	9/24/2010		
Landsat	Operational Land Imager (OLI)	LC81160312013243LGN00	9/19/2014	30 m	0
		LC81160312014214LGN00	9/16/2013		
IKONOS	Panchromatic	IK220020920023325P00	9/20/2002	1 m	0
		IK220020920023325P01			
		IK220020920023325P02			
		IK220020920023408P00			
IKONOS	Multispectral	IK220020920023325M00	9/20/2002	4 m	0
		IK220020920023325M01			
		IK220020920023325M02			
		IK220020920023408M00			
WorldView‐1	Panchromatic		10/9/2009	0.5 m	0
GaoFen‐2 (GF‐2)	Panchromatic and Multispectral CCD Camera Sensors2 (PMS2)	GF2_PMS2_E128.0_N42.0_20170923_L1A0002620874	9/23/2017	0.8 m	0
JiLin (JL‐1)	PMS2	JL1KF01A_PMS03_20210901094039_200059994_101_0016_001_L1_MSS_Clip_90771	9/1/2021	0.4 m	0
		JL1KF01A_PMS03_20210901094039_200059994_101_0016_001_L1_PAN_Clip_90771			
		JL1KF01A_PMS04_20210901094039_200059994_101_0016_001_L1_MSS_Clip_90772			
		JL1KF01A_PMS04_20210901094039_200059994_101_0016_001_L1_PAN_Clip_90772			
Advanced Land Observing Satellite (ALOS)	Panchromatic Remotesensing Instrument for Stereo Mapping (PRISM)	N42080E127826_N41650E128200_LT_DSM		5 m	

### 2.3 Methodology

#### 2.3.1 Extraction of *B. ermanii* Tree Stands

**FIGURE 2:**
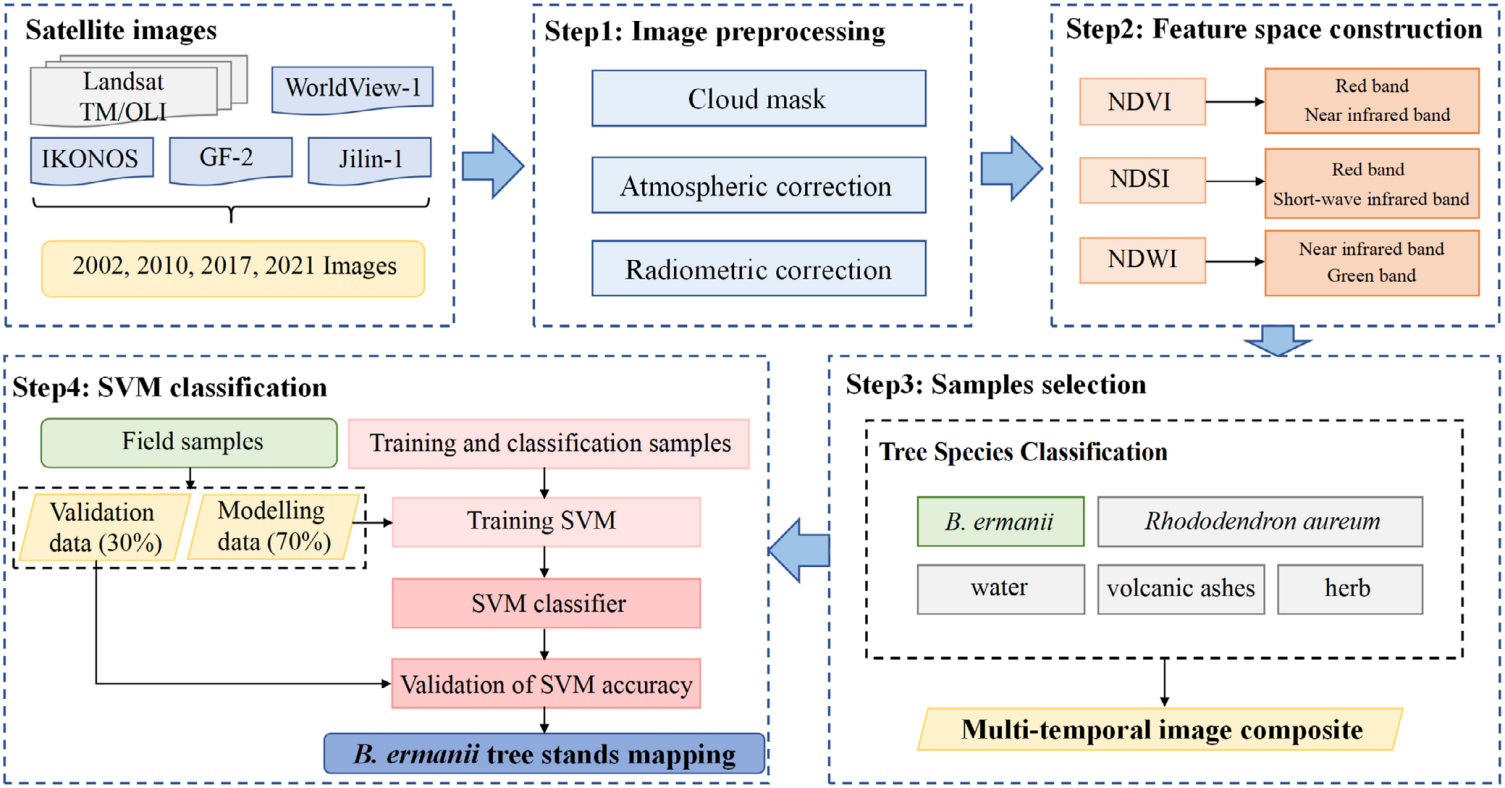
The workflow of the *B. ermanii* classification based on different time‐scale images and SVM algorithm.

The workflow of the *B. ermanii* tree stands classification was shown in Figure 2. Remote sensing images of the Landsat Satellite (20 scenes from 2002 and 2010) and the Gaofen Satellite (six scenes from 2017 and 2021) with no cloud cover were collected to extract *B. ermanii* tree stands in the alpine treeline ecotone on Changbai Mountain across the years 2002, 2010, 2017, and 2021 (Table 1). The high‐resolution remote sensing data used in our study were obtained from a commercial data provider, and these datasets underwent preprocessing, including radiance calibration, atmospheric correction, and image registration. To further ensure geospatial accuracy, we utilized high‐resolution ground control points (GCPs) and a DEM for orthorectification. To enhance the identification capability of spectral features and mitigate the impact of partial environmental factors on classification accuracy, normalized difference water body information (NDWI), normalized difference soil index (NDSI), normalized difference vegetation index (NDVI), and spectral data (Blue, Green, Red, NIR, SWIR1, SWIR2) were used to construct a feature space for classification tasks in the study area. There were nine bands consisting of six reflectance bands and three indices bands with each image. This integration supported the construction of mean and standard deviation for the time‐series data, thereby improving the robustness of the classification process. Furthermore, the distribution range of *B. ermanii* tree stands was estimated and extracted by object‐oriented support vector machine (SVM) algorithm, based on the theoretical framework described by Vapnik (2013). Field vegetation observation samples were used to implement the SVM classification algorithm. Specifically, 70% of the sampling points were used for physical object type classification using the SVM algorithm, while the remaining 30% were reserved for accuracy validation of the classification results. For fine‐scale detail processing, the manual visual interpretation method was applied to comprehensively compare and analyze the fine‐scale patches of *B. ermanii* tree stands across the years 2002, 2010, 2017, and 2021. The interpretation followed a principle that permitted a change in the physical object type of no more than 20%. We defined the classification output as a binary distribution map with a spatial resolution of 50 × 50 m, where a value of 1 represented *B. ermanii* tree stands, and a value of 0 represented non‐*B. ermanii* tree stands. Based on this criterion, patches classified as nonforest type were identified and excluded from the analysis. We collected 480 raw sampling points from field surveys. We initially identified sampling points through visual interpretation of high‐resolution imagery and subsequently validated the presence of *B. ermanii* tree stands with field visits. After removing 39 points located outside the study boundary and 30 points visually interpreted as non‐*B. ermanii* tree stands, 411 points were retained for treeline validation. By overlaying the computer‐interpreted distribution of *B. ermanii* tree stands from 2002, 2010, 2017, and 2020, we identified 147 available sampling points within the *B. ermanii* tree stands distribution range from 2002 to 2021. Among the computer‐interpreted results, 41 points were classified as non‐*B. ermanii* tree stands. Based on the assessment, the overall interpretation accuracy for the *B. ermanii* tree stands distribution in Changbai Mountain from 2002 to 2021 was estimated to be 72.1% (Table 2).

**TABLE 2:** Summary of the number of sampling points, SVM training and validation subsamples, and validation accuracy (%) in this study.

Category	Number of samples/validation accuracy (%)
Raw sampling points	480
Points outside study boundary (removed)	39
Points visually interpreted as non‐*B. ermanii* (removed)	30
Total retained samples	411
Samples for SVM classification (70%)	288
Samples for validation (30%)	123
Samples within *B. ermanii* range (2002–2021)	147
Correctly classified as *B. ermanii*	106
Misclassified as non‐*B. ermanii*	41
Overall classification accuracy	72.1%

#### 2.3.2 Environmental Data

We initially selected 18 environmental factors that may influence *B. ermanii* spatial distribution, including climatic and topographical factors. Twelve climatic variables derived from monthly precipitation and monthly daily maximum, minimum and mean temperatures from CHELSA (https://chelsa‐climate.org) with 30 arc sec spatial resolution (Karger et al. 2017). Topographical variables, such as elevation, slope, aspect, relief and topographic wetness index (TWI), were derived from high resolution DEM (5 m) obtained from the PRISM sensor attached to the ALOS. We used the fishnet tool in ArcGIS 10.2 to divide the study area into 50 m × 50 m grids, generating point data perfectly aligned with the DEM, with each point located at the center of each cell. Then, the ‘Extract Values to Points tool’ was employed to extract corresponding temperature and precipitation data for the points. Pearson's correlation was applied to assess the cross‐correlations, and highly correlated variables (*r* > |0.90|) were eliminated. The threshold minimized multicollinearity, avoiding redundancy that could compromise model accuracy, while retaining relevant information (Schober and Vetter 2020). Only 9 out of the 18 environmental variables were selected based on their relevance and contribution to model performance as evaluator variables (Table 3). To investigate the impact of slope on the distribution of tree species, the slope gradient was extracted at each point and categorized into seven classes by the Geomorphological Survey and Mapping Committee of the International Geographical Society as follows: plain (0%–2%), gently ramp (2%–5%), ramp (5%–15%), steep (15%–25%), very steep (25%–35%), scarp (35%–45%) and vertical slope (> 55%) (Embleton 1981).

**TABLE 3:** Environment factors used for Geodetector in this study.

Factors	Description	Unit
Pre_g_	Precipitation of growing season	mm
Pre_1_	Precipitation of coldest month	mm
Pre_8_	Precipitation of warmest month	mm
*T*_MEAN8_	Mean temperature of warmest month	°C
*T*_MEANg_	Mean temperature of growing season	°C
Elevation	Height above sea level	m
Slope	Relative degree of steepness	°
Relief	Topographic relief degree Max_Regional elevation_ − Min_Regional elevation_ (Niu and Harris 1996)	m
TWI	Topographic wetness index $\ln\left(\frac{\alpha }{\tan\beta }\right)$(Beven and Kirkby 1979)	—

#### 2.3.3 Data Analysis

In order to investigate the environmental factors impacting the changes in the spatial distribution of *B. ermanii*, we employed the Geodetector method to determine the influence of dominant driving factors on its spatial distribution (Chen et al. 2023). The contribution of these factors was quantified using the factor detector, which was calculated by the following equation:

$$\begin{array}{c} q=1-\frac{\sum_{h=1}^{L}N_{h}\sigma_{h}^{2}}{N\sigma^{2}}=1-\frac{\mathrm{SSW}}{\mathrm{SST}}\\ \mathrm{SSW}=\sum_{h=1}^{L}N_{h}\sigma_{h}^{2},\mathrm{SST}=N\sigma^{2} \end{array}$$

where *q* referred to the explanatory power of a single factor on the dependent variable. *h* was the number of strata within variable *Y* or factor *X*. *N*_*h*_ and *N* denoted the number of units in strata *h* and the total number of units across the entire area, respectively. $\sigma_{h}^{2}$ and $\sigma^{2}$ presented the variances of strata *h* and across the entire area, respectively. A higher *q* value represented greater geographical heterogeneity of *Y*. SSW is the sum of squares within the strata *h*. SST is the sum of squares of the entire area. In contrast to traditional regression‐based methods, the Geodetector is more effective at capturing non‐linear interactions and spatial heterogeneity. We also used the interaction detector of Geodetector to evaluate whether the interactions among environmental factors significantly influence the distribution of *B. ermanii*, or if these factors contribute independently (Table S1). By quantifying the interactive and individual effects, this method provides valuable insights into the impact of climatic and topographic variables on treeline dynamics.

## 3 Results

### 3.1 Spatiotemporal Distribution of *B. ermanii* in the Alpine Treeline Ecotone

**FIGURE 3:**
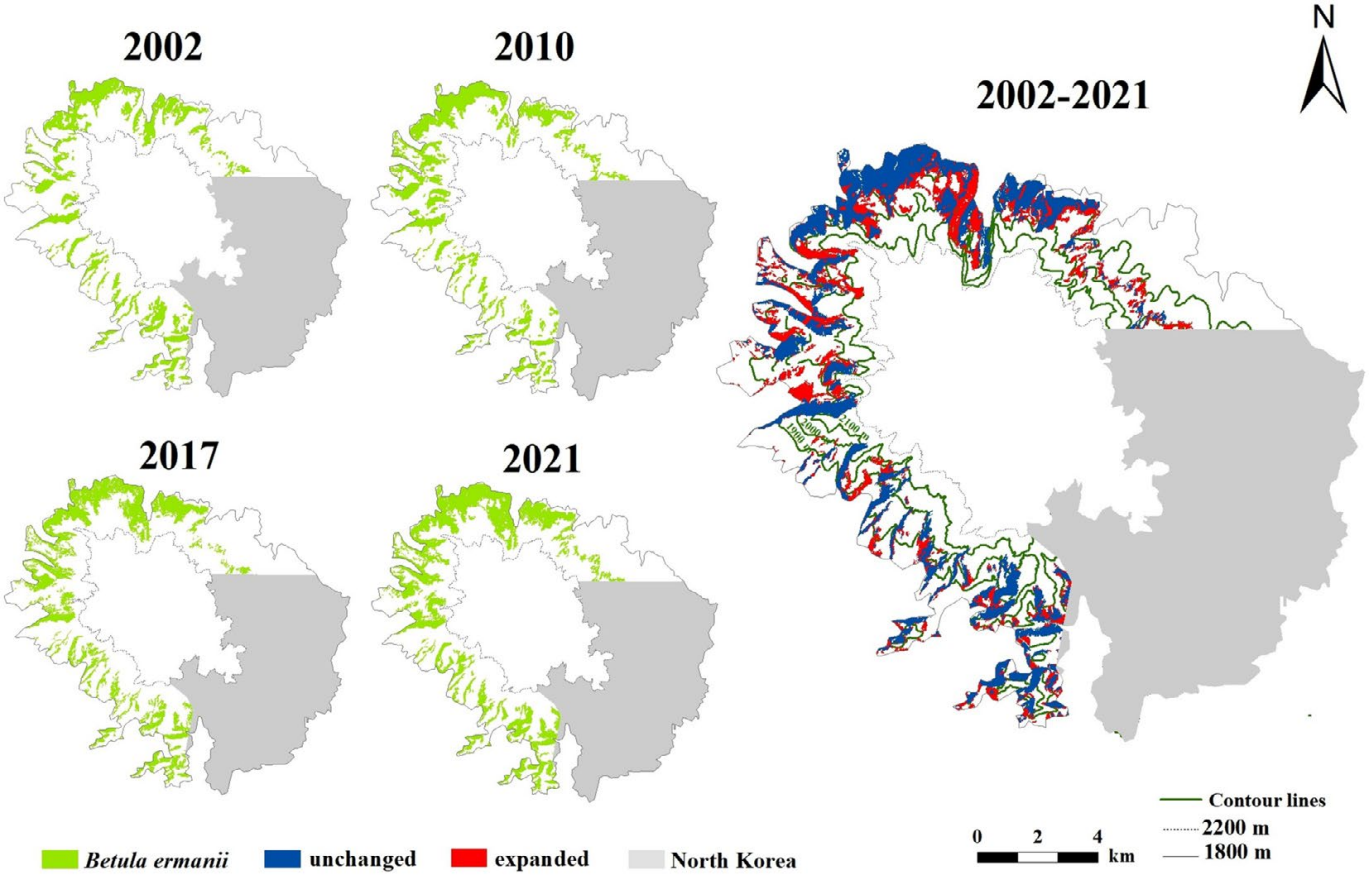
Spatiotemporal distribution of *B. ermanii* in the Changbai Mountains for the years 2002, 2010, 2017, and 2021, along with the dynamics of its distribution changes from 2002 to 2021. No areas of reduction were observed during this period. The study area is between 1800 and 2200 m above sea level (a.s.l.) in Changbai Mountain, excluding North Korea. Blue indicates areas with unchanged distribution, and red highlights areas of expansion.

Overall, *B. ermanii* was the dominant tree species at high elevations, specifically between 1800 and 2200 m, primarily distributed on the northern, western, and northwest sides of Changbai Mountain, covering 77.71% of the study area, excluding North Korea (Figure 3). The distribution of *B. ermanii* showed a decreasing trend with increasing elevation. Over the past two decades, *B. ermanii* expanded by 800.60 hm^2^, reflecting a 24.26% increase compared to its extent in 2002 (Figure 3). From 2002 to 2021, the average annual expansion rate of vegetation was 1.21% (Table 4). However, the rates varied significantly across different historical periods. Initially, there was a decline of 0.71% per year between 2002 and 2010. Subsequently, *B. ermanii* showed an accelerated expansion rate, with increases of 2.33% per year from 2010 to 2017 and 2.38% per year from 2017 to 2021 (Figure 3, Table 4). Overall, the distribution of *B. ermanii* demonstrated an increasing trend across various mountain aspects. The most significant expansions occurred on the western and southern aspects, with rates of 1.99% and 1.00% per year, respectively. In contrast, the expansion on the northern aspect was more modest, with a variation rate of 0.69% per year. Moreover, the rate of upslope expansion was higher across different mountain aspects during 2017–2021 compared to earlier periods. The northern and western aspects showed an overall increase or stability in the maximum elevations of *B. ermanii* distribution over the years. In contrast, the southern aspect demonstrated more variability and a general trend of decrease (Table 5).

**TABLE 4:** Annual expansion rate (% a^−1^) of *B. ermanii* across different mountain aspects over various periods.

Aspects	Periods			
	2002–2010	2010–2017	2017–2021	2002–2021
Northern	−1.22	1.88	2.24	0.69
Western	0.49	2.79	1.90	1.99
Southern	−1.57	2.34	3.56	1.00
Total	−0.71	2.33	2.38	1.21

**TABLE 5:** Maximum elevations (m) of *B. ermanii* distribution by mountain aspects over the years.

Aspects	Year			
	2002	2010	2017	2021
Northern	2200	2122	2176	2223
Western	2217	2194	2228	2224
Southern	2064	2046	2069	2059

**FIGURE 4:**
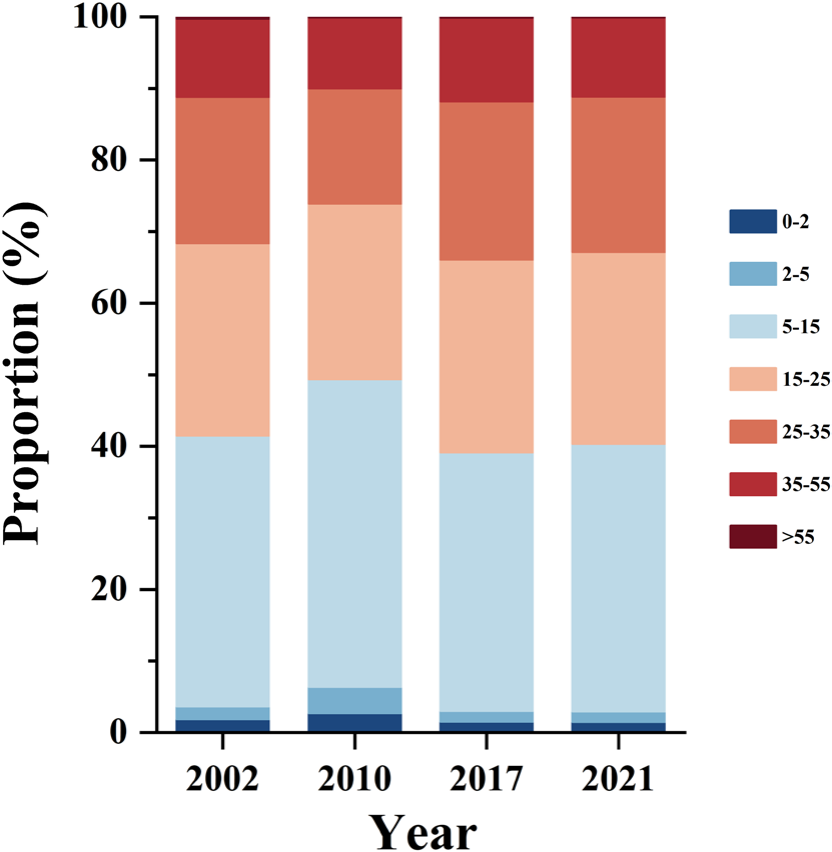
Variation in the distribution proportion (%) of *B. ermanii* across different slopes over various years. Colors represent different slopes.

The area covered by *B. ermanii* initially increased and then decreased with increasing slope steepness. It occupied the largest area on gentle slopes (5°–15°) being the most suitable for its growth (Figure 4). Although the overall distribution area of *B. ermanii* did not vary significantly across different slopes over the years, there was a notable increase over time on steeper slopes (15°–25°, 25°–35°, and 35°–55°) between 2002 and 2021 (Figure 4). However, the distribution on slopes of 5°–15° did not show significant changes over time, indicating that it may have reached saturation in this slope range.

### 3.2 Contribution of Driving Factors to the Expansion of *B. ermanii*

**FIGURE 5:**
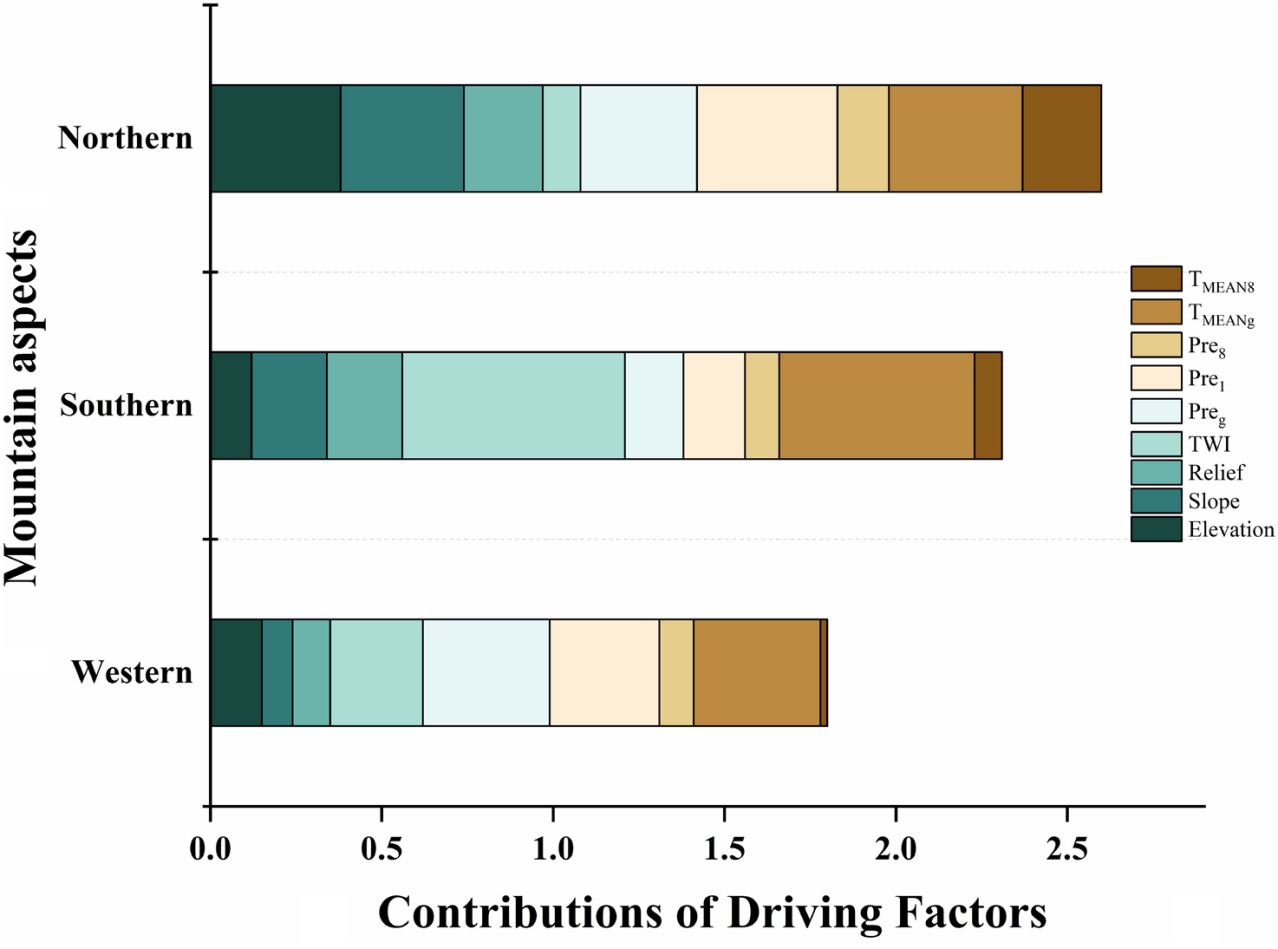
Contributions of driving separate factors to *B. ermanii* distribution across various mountain aspects. Colors represent contributions of different factors, reflecting the combined contributions of multiple factors rather than a normalized value. Results were analyzed by the factor detector of the Geodetector method.

We utilized the factor detector of Geodetector to determine a single dominant factor influencing the increased density of *B. ermanii* across three aspects (Figure 5). Climatic factors were found to significantly influence the increased density on the northern aspect and western aspects, while topographic factors played a more significant role on the southern aspect (Figure 5). Specifically, for the northern aspect, the most influential factors were Pre_1_ (*q* = 0.41), *T*_MEANg_ (*q* = 0.385) and elevation (*q* = 0.383). For the western aspect, the most significant factors were *T*_MEANg_ (*q* = 0.37), Pre_g_ (*q* = 0.367) and Pre_1_ (*q* = 0.319). In the southern aspect, the key factors were TWI (*q* = 0.651), *T*_MEANg_ (*q* = 0.573), slope (*q* = 0.217) and relief (*q* = 0.217). Among the climatic variables, *T*_MEANg_ had the greatest contribution, followed by Pre_1_. Regarding topography factors, TWI was the most influential, followed by slope.

**FIGURE 6:**
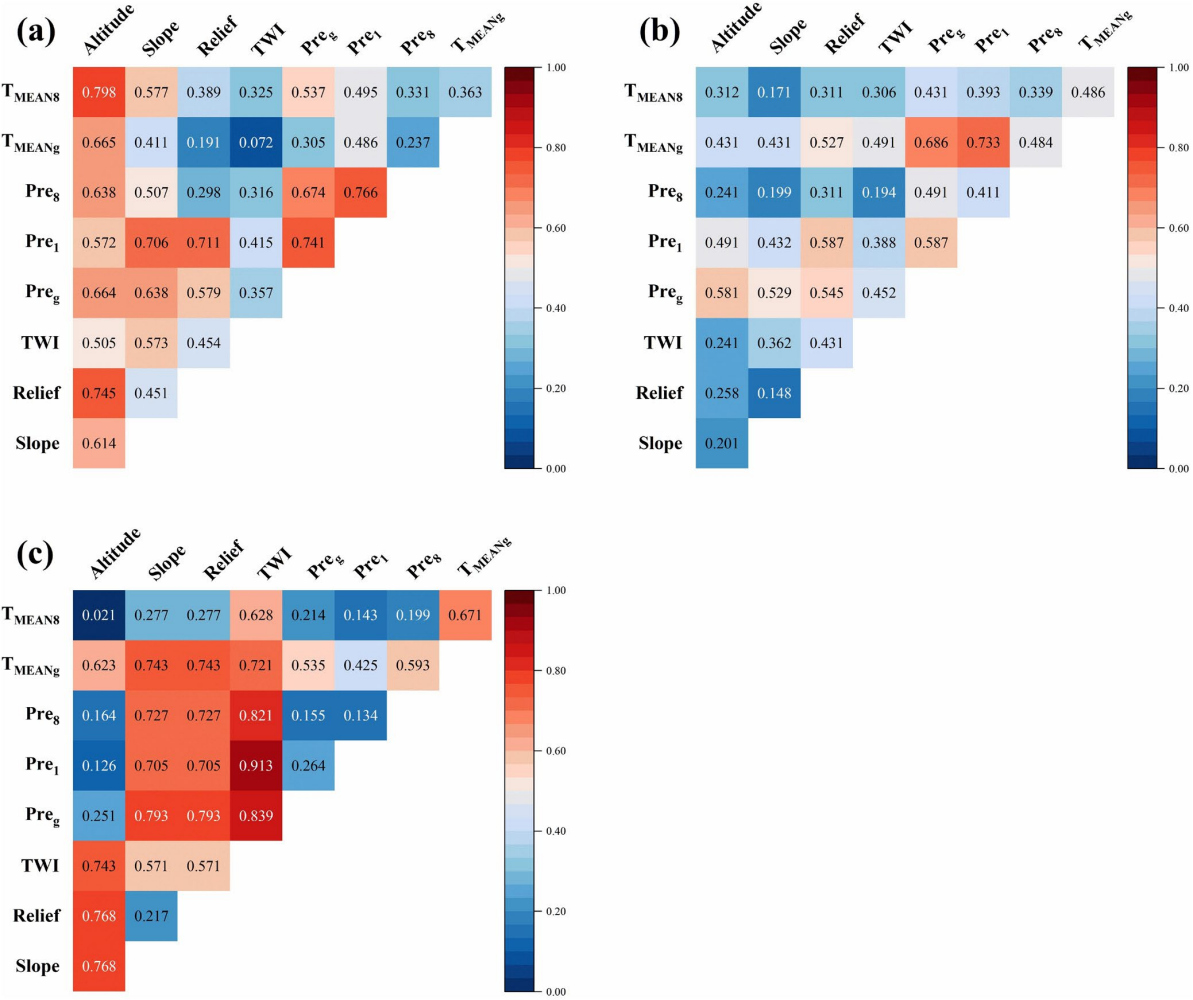
The interactive dominant factors for increased density of *B. ermanii* among various mountain aspects: (a) Northern, (b) Western, and (c) Southern aspects. Colors represent the magnitude of the interaction factor contributions, with red indicating a higher contribution and blue indicating a lower contribution. Results were analyzed using the interaction detector of the Geodetector method. The increased tree density represents the proportion (%) of increased *B. ermanii* area within each grid cell relative to the total grid cell area.

To explore the interactions among these factors, we applied the interaction detector of Geodetector, assessing the dominant interactive factors across three aspects from 2002 to 2021 (Figure 6). We found that multifactor interactions had an enhanced effect on density increase, which could be categorized into two types: dual‐factor enhancement and nonlinear enhancement. Dual‐factor enhancement referred to an interaction where the combined contribution of two factors was greater than the maximum contribution of either factor individually. Nonlinear enhancement, on the other hand, indicated that the interaction between two factors was greater than the sum of the contributions of their individual contributions (Table S1). The interactive factors contributing to the increased density were more pronounced in the northern and southern aspects (Figure 6a,c). In the northern aspect, the dominant interactive factors were *T*_MEAN8_ ∩ elevation (*q* = 0.798), followed by Pre_8_ ∩ Pre_1_ (*q* = 0.766), and relief ∩ elevation (*q* = 0.745) (Figure 6a, Table S1). Meanwhile, the effect of the elevation factor became more pronounced when combined with other factors, contributing more than 50% (*q* > 0.5). In the southern aspect, Pre_1_ ∩ TWI (*q* = 0.913) made a significant contribution, followed by Pre_8_ ∩ TWI (*q* = 0.821), Pre_g_ ∩ slope (*q* = 0.793), and Pre_g_ ∩ relief (*q* = 0.793) (Figure 6c). The interactions between several climatic and topographic factors demonstrated nonlinear enhancement, with contributions exceeding 70% (*q* > 0.7). Comparably, in the western aspect, the *T*_MEANg_ ∩ Pre_1_ had the highest contributions with a *q* value of 0.733, followed by Pre_g_ ∩ *T*_MEANg_ (*q* = 0.686), and Pre_1_ ∩ Relief (*q* = 0.587) (Figure 6b). However, the impact of these dual‐factor interactions was comparatively weaker, with significant contributions mainly attributed to *T*_MEANg_ and Pre_g_ (*q* > 0.5).

## 4 Discussion

### 4.1 Spatial–Temporal Change Patterns of *B. ermanii*

Our results showed that there was a noticeable expansion trend in *B. ermanii* during the last 20 years (Figure 3). Over the past 20 years, the average expansion rate of *B. ermanii* was 1.21% per year, with a particularly rapid increase from 2017 to 2021 (Table 4). This was consistent with the findings by Du et al. (2017) who found that *B. ermanii* was expanding upwards at different rates. Furthermore, changes in the treeline across different aspects were not uniform (Dearborn and Danby 2020; Zheng et al. 2021), which aligned with our findings. Specifically, the expansion rates of *B. ermanii* varied significantly across various mountain aspects, with more rapid expansions on the southern and western aspects and slower expansion on the northern aspects (Table 3). These differences may be attributed to microclimates in mountain areas. Different aspects received varying amounts of solar radiation and were subjected to different wind conditions (Thornton et al. 2021; Li et al. 2022), leading to varied moisture and thermal environments, which affected the distribution and growth of plants (Ferrer‐Castán and Vetaas 2005; Aqeel et al. 2023). Numerous studies confirmed that tree distribution decreased with increasing elevation (Wang et al. 2017; Mu et al. 2022; Kienle et al. 2023), and these patterns were consistent with our findings. The distribution of *B. ermanii* decreased with increasing elevation, ranging from 2000 to 2200 m (Figure 3). The maximum elevation distribution of *B. ermanii* was higher on the western and northern aspects, exceeding 2200 m, whereas on the southern aspect, the highest elevation was relatively lower, at only 2059 m. Our results were consistent with those of Du et al. (2017), who reported that *B. ermanii* reached peak elevations of over 2140 and 2200 m on the northern and western aspects, respectively (Table 5). On one hand, the higher elevations of the western and northern mountain aspects provide more space for the upward expansion of trees (Holtmeier and Broll 2019). On the other hand, these differences in elevation also result in varying levels of solar radiation, which subsequently influence temperature and precipitation conditions (Rita et al. 2023). Treeline limitations on the northern aspect may be primarily driven by temperature, particularly during the summer months, as indicated by the high correlation with summer temperatures (Figure 6a). In contrast, the treeline on the southern aspect appeared to be more influenced by precipitation and TWI, while on the western aspect, precipitation and growing season temperature played a more significant role, as these variables showed greater significance for these mountain aspects (Figure 6c).

Our study showed that the distribution of *B. ermanii* trees also varied with slope degrees. We found that *B. ermanii* was most abundant on gentle slopes ranging from 5° to 15° and least abundant on steep slopes exceeding 55° (Figure 4). Although the distribution on slopes of 5°–15° remained stable over time, there was a notable increase on steeper slopes (15°–25°, 25°–35°, and 35°–55°) (Figure 4). To adapt to the low temperatures and strong winds at high elevations, trees at the treeline sometimes referred to as krummholz, exhibited a dwarf, crooked growth form (Körner 1999). Gentle slopes could accumulate sufficient snow cover, which ensures that soil temperature beneath the snow remains higher than that above it. This suggests that snow cover effectively delays soil freezing, thereby protecting treeline trees from extreme cold and enabling their survival throughout the long winter season (Holtmeier 2009). Snow cover provides insulation against extreme temperatures, and gentle slopes create favorable microclimatic conditions, contributing to the formation of “Microrefugia” for species. Additionally, the increasing abundance of *B. ermanii* on steeper slopes may indicate an adaptation strategy to climate changes. Steeper slopes are harsher environments due to thinner soils, reduced water retention, and greater temperature variations (Norton and Von Blanckenburg 2010). However, by colonizing these areas, *B. ermanii* experiences reduced competition from other species, as fewer plants can tolerate such challenging conditions. This adaptation enables *B. ermanii* to expand its range into harsher environments, thereby enhancing its resilience to climate change.

### 4.2 Topography and Climatic Influence on Density Change for *B. ermanii*

We used the Geodetector method to analyze the distribution differences of *B. ermanii* across different aspects of the Changbai Mountain, revealing the combined effects on treeline shifts. An earlier study identified climate warming, rather than nutrient availability, as the primary driver of the upward migration of *B. ermanii* in the Changbai Mountains (Du et al. 2017). Our results further showed that the interaction between multiple factors had a greater impact on the expansion of *B. ermanii* than any single dominant factor (Figures 5 and 6). These findings highlight the importance of accounting for both climatic and topographic influences in conservation strategies, as understanding treeline dynamics is crucial for preserving biodiversity in response to climate change. Notably, the increased density of *B. ermanii* showed different combined interactions across different aspects. The differentiation between northern and western aspects was important, as these orientations experience different microclimatic conditions that affect growth and expansion rates. Elevation and its interaction with climatic factors dominated the northern aspect, contributing over 50% (Figure 6a). Specifically, on the northern aspect, the interaction between elevation and mean temperature of the warmest month had the most significant impact on the expansion of *B. ermanii* (Figure 6a). Elevation gradients played a critical role in *B. ermanii* expansion by influencing key climatic conditions, such as temperature and precipitation. Cooler temperatures and variable precipitation at higher elevations impact the physiological processes of *B. ermanii*. Temperature variations along elevation gradients determine growing season length, directly affecting tree establishment and growth rates (Zou et al. 2022). Additionally, higher elevations often receive more precipitation, supporting soil water availability and promoting vegetation growth (Wang, Mao, et al. 2021). However, the western aspect was primarily influenced by the interaction between mean temperature of the growing season and precipitation of the coldest month (Pre_1_) (Figure 6b). Similarly, in the Valtellina region of the Italian Alps, treeline expansion is driven by climate, topography, and minimal human influence (3%), with the western aspect primarily shaped by climate factors, consistent with our findings (Leonelli et al. 2016). Rising summer temperatures relieve the temperature limitation, facilitating treeline trees growth and promoting their upward migration into tundra areas (Andreu‐Hayles et al. 2020; Rees et al. 2020). Wang et al. (2017) confirmed that the mean temperature of the growing season showed a significant increasing trend in the tundra zone of the Changbai Mountain, suggesting that adequate thermal conditions are essential for the expansion of vegetation. Warming promoted the expansion of *B. ermanii* in Changbai Mountain, with precipitation modifying this expansion as climate change continues (Cong et al. 2024). The interaction between temperature and precipitation played a significant role in determining the future potential distribution of *B. ermanii* within the alpine treeline ecotone. However, the reduction of the tundra zone may lead to a decline in specialized cold‐adapted species, reducing overall biodiversity. As trees encroach into tundra areas, habitat structure will change, affecting both species that adapt to new conditions and those reliant on tundra environments.

Previous studies have shown that at high elevations, the growth of *B. ermanii* is significantly correlated not only with mean temperature but also with precipitation during the growing season (Wang et al. 2013). Moreover, studies on the Khibiny Massif and the western Putorana Plateau suggested snow played a critical role in promoting treeline expansion on southern slopes (Grigoriev et al. 2022). However, insufficient snow cover may cause winter freeze–thaw cycles that adversely affect seed germination (Shakhmatov et al. 2022). Snow, as precipitation during the coldest month, provides an insulating layer for vegetation (Peng et al. 2024), which alleviates the decrease in temperature and maintains soil temperature necessary for seedlings survival at the beginning of the growing season (Hagedorn et al. 2014). Snowfall also contributes to soil moisture retention, which is crucial for supporting plant growth during the following growing season, especially under the harsh environmental conditions (George 2014). The beneficial effects of snowfall are primarily attributed to the snowpack's thermal insulation, which leads to higher winter soil surface temperatures, enhancing decomposition and nutrient release (Baptist et al. 2010; Hallinger et al. 2010). These favorable environmental conditions collectively promote the growth of saplings and seedlings.

The expansion of *B. ermanii* on the southern aspect was primarily driven by topographical factors, with the interaction between the TWI and other factors contributing over 60% (Figure 6c). The interaction of TWI and Pre_1_ was especially high; the contribution is 91%. TWI may be related to volcanic activity since the southern aspect of Changbai Mountain is covered with thick layers of volcanic ash and pumice (Jin et al. 2013). *B. ermanii* trees are primarily distributed in areas with slopes of 5°–15°, but their distributions are negatively correlated with increasing slope. This was consistent with the findings by Guo (2015) who found that in areas with gentler slopes and lower elevations, accumulated soil can create a stable geological foundation that supports vegetation growth, thus promoting expansion into the tundra. Wang, Shen, et al. (2021) suggested that precipitation in the southern region of Changbai Mountain has shown a nonsignificant decreasing trend from 1961 to 2018. Evidence from studies in Eurasia and the Americas indicated that, in addition to climate warming, treeline expansion was significantly influenced by other environmental factors such as wind and topography (Camarero et al. 2021). Strong winds on the southern aspect of Changbai Mountain reduce local moisture and cause subsidence, leading to a decline in precipitation (He et al. 2020). Excessive rainfall can lead to erosion of the loose soil formed by volcanic ash and pumice, which hinders vegetation succession (Jin et al. 2013). Therefore, the expansion of *B. ermanii* on the southern aspect can be attributed to suitable precipitation and stable soil moisture conditions.

A number of other factors not considered in this study may contribute to uncertainty in our study. We only simulated the effects of climate change and topographic variables on the distribution of treeline trees. We identified climatic and topographic factors as the primary climatic factors affecting tree species, without considering species competition and disturbance processes, which can also significantly impact tree species distributions. Nevertheless, we also acknowledge that differences in spatial resolution could introduce uncertainties in our findings. However, our analysis was supported by satellite data validated with ground‐truth data collected during field surveys, which allowed for a more robust and accurate identification of treeline positions. Despite such limitations, there are good reasons to believe that our approach effectively assesses how climate change and topographic variables interact to affect tree species distributions.

## 5 Conclusion

This study demonstrated that both climatic and topographic factors were crucial in determining the dynamics of tree species distribution in the alpine treeline ecotone of the Changbai Mountain. Consistent with our first hypothesis, we found that the expansion rate of *B. ermanii* in the alpine ecotone of the Changbai Mountain in China varied with mountain aspect slopes. Furthermore, the combined influence of climatic and topographic factors on this expansion confirmed our second hypothesis that multifactorial drivers, including climate and topography, shaped the dynamics of *B. ermanii* distribution in these ecotones, despite these effects differing significantly among the mountain aspects. Topographic complexity not only strongly affects vegetation expansion, but also provides suitable habitats enhancing species survival and colonization under climate change. Therefore, our study highlights the necessity of considering multiple environmental variables to understand tree species distribution in the alpine treeline ecotone and to forecast treeline dynamics in response to global climate change.

## Author Contributions

**Yingyi Chen:** data curation (equal), formal analysis (lead), investigation (lead), writing – original draft (lead). **Yongfeng Gu:** formal analysis (supporting), software (lead), validation (lead), visualization (lead). **Wen J. Wang:** software (equal), validation (equal), visualization (equal), writing – review and editing (equal). **Lei Wang:** software (equal), validation (equal), visualization (equal), writing – review and editing (equal). **Xiaodong Li:** software (equal), validation (equal), visualization (equal), writing – review and editing (equal). **Shengwei Zong:** software (equal), validation (equal), visualization (equal), writing – review and editing (equal). **Mai‐He Li:** writing – review and editing (equal). **Zhengfang Wu:** writing – review and editing (equal). **Hong S. He:** writing – review and editing (equal). **Yu Cong:** conceptualization (equal), funding acquisition (lead), methodology (equal), resources (lead), supervision (equal), writing – review and editing (lead). **Ming Jiang:** conceptualization (equal), funding acquisition (equal), methodology (equal), resources (equal), supervision (equal), writing – review and editing (equal).

## Conflicts of Interest

The authors declare no conflicts of interest.

## Supporting information


Table S1

## Data Availability

The original data in this manuscript are available in the Figshare data repository (https://figshare.com/s/33e507f2f84861e67409). As the interpretation and mapping presented in this study were performed using ArcGIS, ENVI, and Origin software, no custom code was utilized.
