# Supplementary material for: Climate Change and Topography Drive the Expansion of Betula ermanii in the Alpine Treeline Ecotone of the Changbai Mountain
Source: Ecol Evol. 2025 May 8;15(5):e71368. doi: 10.1002/ece3.71368 (PMC12061449; doi:10.1002/ece3.71368)
Supplement: Supplementary file 1 — Table S1 [file ECE3-15-e71368-s001.docx]

**Supplementary Material**

**Table S1.** Classification of the interaction types between two independent variables in Geodetector analysis.

| q value | Interaction | Instruction |
| --- | --- | --- |
| q(*X_1_*∩*X_2_* ) < *Min* (q(*X_1_*),q(*X_2_*)) | Nonlinear weaken | q(*X_1_*∩*X_2_*) means the interaction between q(*X_1_*) and q(*X_2_*)  *Min* (q(*X_1_*),q(*X_2_*)) means take the minimum value between q(*X_1_*) and q(*X_2_*)  *Max* (q(*X_1_*),q(*X_2_*)) means take the maximum value between q(*X_1_*) and q(*X_2_*)  q(*X_1_*) + q(*X_2_*) means to sum q(*X_1_*) and q(*X_2_*) |
| *Min* (q(*X_1_*),q(*X_2_*)) < q(*X_1_*∩*X_2_*) < *Max* (q(*X_1_*),q(*X_2_*)) | Nonlinear weaken for single factor |  |
| q(*X_1_*∩*X_2_*) > *Max* (q(*X_1_*),q(*X_2_*)) | Dual-factor enhance |  |
| q(*X_1_*∩*X_2_*) = q(*X_1_*) + q(*X_2_*) | Independence |  |
| q(*X_1_*∩*X_2_*) > q(*X_1_*) + q(*X_2_*) | Nonlinear enhance |  |
